# Antarctic aldehyde dehydrogenase from *Flavobacterium* PL002 as a potent catalyst for acetaldehyde determination in wine

**DOI:** 10.1038/s41598-022-22289-8

**Published:** 2022-10-15

**Authors:** V. I. Paun, R. M. Banciu, P. Lavin, A. Vasilescu, P. Fanjul-Bolado, C. Purcarea

**Affiliations:** 1 Department of Microbiology, Institute of Biology, 296 Splaiul Independentei, 060031 Bucharest, Romania; 2 International Centre of Biodynamics, 1B Intrarea Portocalelor, 060101 Bucharest, Romania; 3 Departamento de Biotecnología, Facultad de Ciencias del Mar y Recursos Biológicos, Universidad de Antofagasta, 1240000 Antofagasta, Chile; 4 Metrohm DropSens, S.L.,Vivero de Ciencias de la Salud, C/Colegio Santo Domingo de Guzmán s/n, 33010 Oviedo, Asturias Spain

**Keywords:** Biochemistry, Biotechnology

## Abstract

Latest solutions in biotechnologies and biosensing targeted cold-active extremozymes. Analysis of acetaldehyde as a relevant quality indicator of wine is one example of application that could benefit from using low-temperatures operating catalysts. In search of novel aldehyde dehydrogenases (ALDH) with high stability and activity at low temperatures, the recombinant S2-ALDH from the Antarctic *Flavobacterium* PL002 was obtained by cloning and expression in *Escherichia coli* BL21(DE3). Structural and phylogenetic analyses revealed strong protein similarities (95%) with psychrophilic homologs, conserved active residues and structural elements conferring enzyme flexibility. Arrhenius plot revealed a conformational shift at 30 °C, favoring catalysis (low activation energy) at lower temperatures. In addition to a broad substrate specificity with preference for acetaldehyde (Km = 1.88 mM), this enzyme showed a high tolerance for ethanol (15%) and several salts and chelators (an advantage for wine analysis), while being sensitive to mercury (I_50_ = 1.21 µM). The neutral optimal pH (7.5) and the stability up to 40 °C and after lyophilization represent major assets for developing S2-ALDH-based sensors. An enzymatic electrochemical assay was developed for acetaldehyde detection in wines with proven accuracy in comparison with the reference spectrophotometric method, thus evidencing the potential of S2-ALDH as effective biocatalyst for industry and biosensing.

## Introduction

Aldehydes are volatile, reactive carbonyl compounds, widely used in the food and cosmetics industries as important contributors to emerging olfactory technologies. This class of chemical compounds is relevant for assessing the human health status and used as indicators of the quality of environmental air^1^, food^2^ and beverages^3^. Among these compounds, acetaldehyde is known to affect the color, stability and aroma of alcoholic beverages^3^. This aldehyde is present in wine at concentrations up to 211 mg/L, while it could reach 63 mg/L and 1159 mg/L in beers and spirits, respectively^4^. In wine, acetaldehyde is formed during alcoholic fermentation, and also produced in later stages by acetic bacteria. This compound binds to bisulfite used as stabilizer, thus preventing its antimicrobial and antioxidant actions^5^. Moreover, acetaldehyde forms bridges between polyphenolic compounds such as flavanols and anthocyanins (e.g. catechin and malvidin-3-*O*-glucoside) with formation of oligomeric compounds responsible for the change of color and aroma in wines^6^. Aldehydes bound in adducts with bisulfite can be liberated during the wine ageing when the sulfite gets oxidized, thus the dynamics of this class of reactive carbonyl compounds critically influences the wine organoleptic characteristics^7^. In addition to acetaldehyde, other aldehydes present at µg/L concentrations contribute to wine aroma, counting isovaleraldehyde, isobutyraldehyde, 2-methylbutanal, octanal, nonanal, decanal, phenylacetaldehyde and benzaldehyde^7,8^.

In liquid phase, the aldehydes content could be measured by a variety of methods counting chromatography with different detection modes (UV–VIS spectrometry, fluorescence, mass spectrometry), titration, and enzymatic assays^3,9,10^. In these cases, specific detection requires separation and quantitation of individual aldehydes. In addition to the time consuming and complexity of many tests, they require expensive laboratory equipment. Alternatively, the level of aldehydes in beverages, food products, and pharmaceutical ingredients could be measured using enzyme-based biosensors relying on various enzyme such as aldehyde dehydrogenases (ALDH) from different sources, aldehyde oxidoreductase PaoABC from *E. coli*, or alcohol dehydrogenases (ADH) based on the reverse reaction^11–18^. The main advantages of biosensors as compared to the classical analytical methods reside on their ability for real-time measurements, being compatible with portable cost effective equipment for on-site measurements.

Aldehyde dehydrogenase superfamily catalyzes the NAD(P)^+^-dependent oxydation of highly reactive aliphatic and aromatic aldehydes to the corresponding carboxylic acid^19,20^. Monitoring the aldehydes concentration relies on the quantification of NADH^+^ formed during the enzymatic reaction, and is commonly carried out by spectrometry and fluorimetry^20^. In comparison with these methods, electrochemical assays and biosensors utilization provided several advantages related to their reduced cost and portability. While the field of biosensing advanced considerably towards miniaturized and user friendly transducer devices^21^, the search for new enzymes with improved characteristics including variable substrate specificity and high catalytic efficiency for targeted substrates, lack of sensitivity to ions and compounds typically found in real samples, and high stability and enzymatic activity in a wide range of temperatures is still ongoing^22^.For the last decades, microbial catalysts were extensively used for a variety of industrial and biosensing applications, most of them originating from mesophilic strains^23^. More recently, extremophilic microorganisms thriving under extreme condition of temperature, salinity, pH, hydrostatic pressure, radiation, etc.^24–27^ showed a rapid development in using their molecules adapted to cope with a wide range of biophysical parameters^28,29^. Among extremophilic microorganisms hosting these potent enzymes (extremozymes), thermophilic and hyperthermophilic strains isolated from hot environments were among the main source of industrial catalysts due to their high stability and distinctive substrate utilization profile^30,31^.

Alternatively, cold-active enzymes were actively screened for a wide range of applications in^32–36^, being active at low temperatures and presenting different substrate specificity as compared to enzymes from mesophilic and thermophilic counterparts^37,38^. Our group has previously reported the production and characterization of a novel cold-active recombinant aldehyde dehydrogenase from the Antarctic *Flavobacterium* PL002, highlighting its applicatve potential for the electrochemical detection of benzaldehyde in pharmaceutical ingredients^15,39,40^. However, to date, limited data on cold-active oxidoreductases is available for developing catalysts for industrial applications^36,41^.

In this context, the current study reported the cloning and characterization of a new recombinant aldehyde dehydrogenase from the Antarctic *Flavobacterium* PL002 strain (S2-ALDH) as a valuable bacterial cold-active catalyst for measuring acetaldehyde in liquid phase, highlighting for the first time the enhanced characteristics of a cold-active-ALDH-based assay for measurements in wine. Specifically suitable for this application, S2-ALDH enables measurements at wine cellar temperatures and is not sensitive to ethanol and phenolic compounds. The assay uses electrochemical detection based on screen-printed carbon nanotubes electrodes as transducers. The accuracy of the new system was confirmed in comparison with a reference spectrophotometric method.

## Results and discussion

### Sequence analysis of S2-ALDH

BLAST screening of the *Flavobacterium* PL002 genome sequence^42^ led to identification of a coding region homologous to aldehyde dehydrogenase gene (1365 bp) coding for the hypothetical enzyme S2-ALDH of 454 amino acids, with calculated MW 49,062 (Supplementary Fig. 1S).

Phylogenetic analysis of the S2-ALDH amino acid sequence in relation with aldehyde dehydrogenases from genus *Flavobacterium* showed three distinct clusters mainly composed of psychrophilic and psychrotolerant species (Supplementary Fig. 2S). Among these, Clade I (bootstrap = 98) comprised one strain from Arctic soil (WP132111999) and an Antarctic sub-clade (bootstrap 94) composed of three *Flavobacterium* strains originating from sea ice (WP091086047, WP074724235, WP101136856), and from a meromictic lagoon (WP016989072). Clade II (bootstrap 100) also comprised Antarctic strains isolated from sea ice (WP 007139642) and lake microbial mat (WP091433283), while Clade III (bootstrap 99) was composed of Antarctic marine strains. Within the latter one, S2-ALDH showed high similarity (> 99%) with closely related sequences of an Antarctic unclassified *Flavobacterium* strain (WP173857136) (100%) and *Flavobacterium* 28A (WP173851899) (99%), clustering with the well supported sub-clade (bootstrap 86) of the psychrophilic *F. faecale* (WP108740064) isolated from Antarctic penguin feces and *F. frigidarium* (WP026707351) retrieved from Antarctic marine sediment, and with the psychrotolerant *F. kayseriense* (WP187011286) originating from a farmed rainbow trout (Supplementary Fig. 2S).

Pair alignment of the S2-ALDH amino acid sequence with homologous enzymes from psychrophilic, mesophilic and (hyper)thermophilic species (Table 1) confirmed the strong resemblance with the psychrophilic enzyme, and highlighted relatively low similarity and identity percentages with ALDHs from species thriving at higher temperatures. However, their sequence identity showed an overall descending trend with the increase of the environmental growth temperature of the host, varying from 90.1% (psychrophiles) to 30.2–40.2% (mesophiles) and 28–33.3% (hyper/thermophiles) (Table 1), in support of the presence of common structural features of cold-adapted enzymes^43^.

**Table 1:** Sequence identity and similarity of S2-ALDH with homologous ALDHs from psychrophilic (*Flavobacterium frigidarium)*, mesophilic (*Escherichia coli* and *Staphylococcus aureus)* and (hyper)thermophilic (*Thermus thermophilus* and *Sulfurisphaera tokadaii str. 7)* strains.

*F. frigidarium* WP026707351		*E. coli* WP089574674		*S. aureus* WP000421701		*T. thermophilus* WP011173038		*S. tokodaii str. 7* BAB65021		
% id	% sim	% id	% sim	% id	% sim	% id	%sim	% id	% sim	
90.1	94.9	40.2	59.4	30.2	48.5	28.0	47.3	33.3	52.6	S2-ALDH
		39.5	60.3	30.4	50.2	28.0	47.6	33.5	55.1	*FF*
				26.5	47.7	28.5	44.2	29.1	49.5	*EC*
						33.0	52.8	31.3	50.4	*SA*
								30.8	53.5	*TT*

The aminoacid composition of this Antarctic enzyme indicated a lower number of cysteine residues as compared to that of mesophilic ALDHs, that could induce a higher protein flexibility at low temperatures by a reduced disulfide bond formation^44^, similar to homologous enzymes from the analyzed thermophilic and hyperthermophilic species (Supplementary Table 1S). Meanwhile, the low content of prolines that could rigidify flexible regions^45^ as compared to ALDHs from both mesophilic^46,47^ and (hyper)thermophilic^48,49^ species could also contribute to enhance enzyme flexibility at low temperature. The total number of positively (Arg + Lys) and negatively (Asp + Glu) charged residues of S2-ALDH occupied an intermediary position between that of the *E. coli* and *T. thermophilus* ALDHs (Supplementary Table 1S), suggesting improved ion interactions relative to the mesophilic enzyme, but reduced as complared with the thermophilic ones. Considering the ratio of Arg/(Arg + Lys) residue numbers, where a relatively low value suggests a low presence of ion interactions^50^, the estimated salt bridges formed in S2-ALDH (0.2692) appeared to be reduced as compared to that of mesophilic (0.3396–0.5870) and (hyper)thermophilic (0.4355–0.6557) homologous enzymes (Supplementary Table 1S), in support of a higher flexibility in the case of the cold-active extremozymes to cope with low temperature catalysis^43^.

The multiple alignment of these ALDHs (Supplementary Fig. 3S) revealed the presence of all catalytic residues universally conserved, Asn131, Glu228, Gly259 and Cys262 (numbers in S2-ALDH), in support of a functional Antarctic enzyme. Moreover, the substrate binging site of S2-ALDH was highly conserved among the investigated homologous enzymes, comprising identical (Pro129, Lys154, Thr205, Gly206, Leu229, Gly230, Glu359, Phe361), and partially conserved (Trp130, Leu204, Ala210, Met214, Phe425) residues. Among these, all the NAD^+^ cofactor binding sites Trp130, Lys154, Gly206, Glu359 and Phe361 were fully conserved. Based on sequence homology with *S. aureus* ALDH^51^, the oligomerizations domains (Phe101-Gly122) and (Asn447-Ser454) exhibited a partial conservation, in particular between the enzymes originating from psychrophilic hosts (Supplementary Fig. 3S). The Phe101-Gly122 region showed a high content of Ser (13.6%) and Thr (17.4%) residues for S2-ALDH and *F. frigidarium* ALDH, respectively, while charged residues (13–13.6%) and hydrophobic residues Leu, Ile, Val (13.6%) were highly represented in the mesophilic and thermophilic counterparts. The Asn447-Ser454 stretch was characterized by a high content of positively charged residues (37.5%) in comparison with mesophilic enzymes from *E. coli* (20%) and *S. aureus* (9.7%), and displayed close values of hydrophobic residue content (37.5%) with those of mesophilic enzymes (31.6%-40%), while significantly reduced relative to that of the hyperthermophilic enzyme (60%) (Supplementary Fig. 3S).

### Cloning, expression and purification of the cold-active enzyme

The S2-ALDH gene encoding the hypothetical S2-ALDH *Flavobacterium* PL002 aldehyde dehydrogenase was synthesized and inserted into the pHAT2 expression vector using NcoI/BamHI cloning sites (ATG: Biosynthetics GmbH, Merzhausen, Germany), in order to append a His-tag to the amino end of the recombinant protein and facilitate purification. The heterologously expressed enzyme in *Escherichia coli* BL21 (DE3) was purified from the soluble fractions in a single step by Ni^2+^ affinity chromatography, with a yield of 1.02 ± 0.12 µg S2-ALDH /L culture (Supplementary Fig. 4S). The specific activity of the purified enzyme measured at 25 °C in the presence of 1 mM acetaldehyde and 10 mM NAD^+^ cofactor was of 0.51 ± 0.15 U/mg.

### Biochemical characterization of the recombinant S2-ALDH

**Figure 1:**
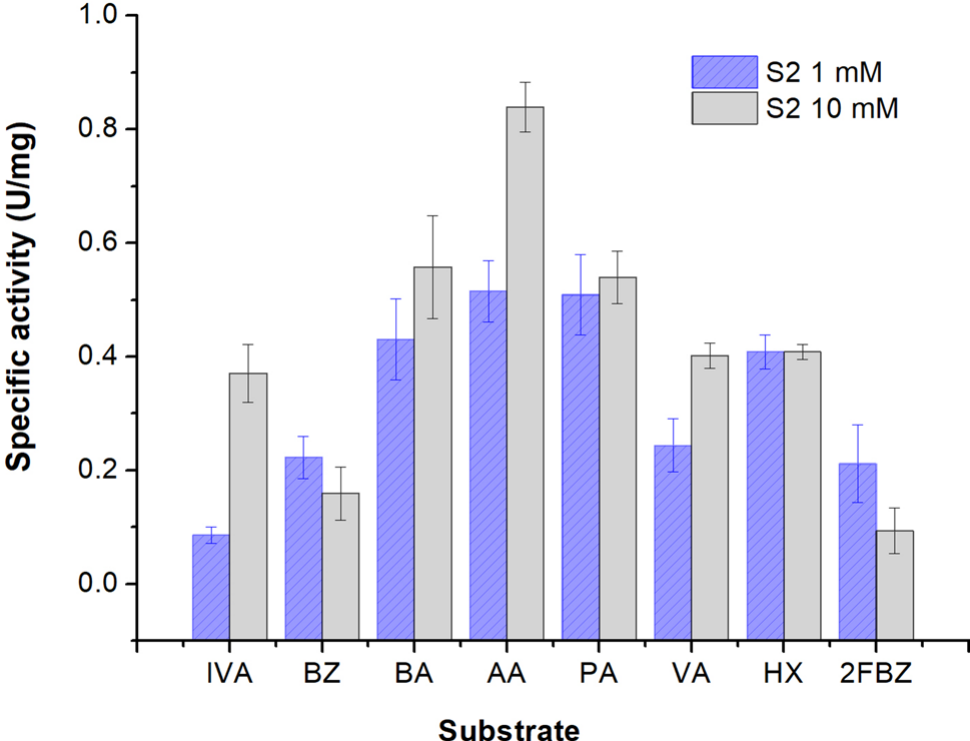
Substrate specificity of S2-ALDH. The specific activity of S2-ALDH was measured at 25 °C as indicated in “Methods”, using 1 mM (purple) and 10 mM (grey) aliphatic aldehydes isovaleraldehyde (IVA), butyraldehyde (BA), acetaldehyde (AA), propionaldehyde (PA) and valeraldehyde (VA) and aromatic aldehydes (benzaldehyde (BZ) and 2-fluorobenzaldehyde (BZ) as substrates, in the presence of 1 mM NAD^+^.

*Substrate specificity.* The catalysis of different aliphatic and aromatic aldehydes by this cold-active dehydrogenase showed a variable substrate specificity in the presence of both high and low concentrations of aldehydes (Fig. 1).

When using 10 mM aldehydes, the enzyme showed a preference for saturated aliphatic aldehydes, with the highest activity measured for acetaldehyde (0.84 U/mg), while at lower substrate concentration (1 mM), both acetaldehyde and propionaldehyde provided the highest S2-ALDH activity (0.51 U/mg), followed by hexanal (0.41 U/mg) and butiraldehyde (0.43 U/mg). In the presence of 10 mM substrate, the aliphatic aldehydes isovaleraldehyde, valeraldehyde and hexanal showed comparable activities. Noteworthy, the unsaturated aliphatic aldehyde hexanal appeared to be a potent substrate for S2-ALDH at both low and high concentrations. Meanwhile, isovaleraldehyde was one of the substrates leading to the lowest activity when tested at low concentration (1 mM). Also, the aromatic aldehydes benzaldehyde and 2-fluorobanzaldehyde exhibited low ALDH activity at both 1 mM and 10 mM concentrations (Fig. 1). Substrate specificity of S2-ALDH was different from that of the aldehyde dehydrogenase from the same Antarctic microorganism which preferred isovaleraldehyde among the aliphatic aldehyde substrates^39^, and also different from that of the commercially available mesophilic aldehyde dehydrogenase from yeast^52^ and human^53^. Thus, the preference for acetaldehyde and the different substrate specificity indicated S2-ALDH as a promising component of bioelectronic e-tongues, i.e. arrays of enzyme biosensors, each modified with a different enzyme with slightly different and overlapping specificities. Such arrays are used to analyze a mixture of enzymatic substrates, enabling a specific and quantitative response of all or some of the substrates via a chemometric method^54^.

*Optimal pH* established for the NAD+-dependent reaction of propionaldehyde in the presence of various buffers covering the pH 6.0–10.5 interval (see “Methods”) indicated the highest activity of S2-ALDH when using 100 mM potassium phosphate pH 7.5 as buffer (not shown). This high activity at neutral pH is advantageous for biosensing applications that include electrochemical mediators, as well as for coupled cascade enzymatic reactions with enzymes having similar optimum pH.

**Figure 2:**
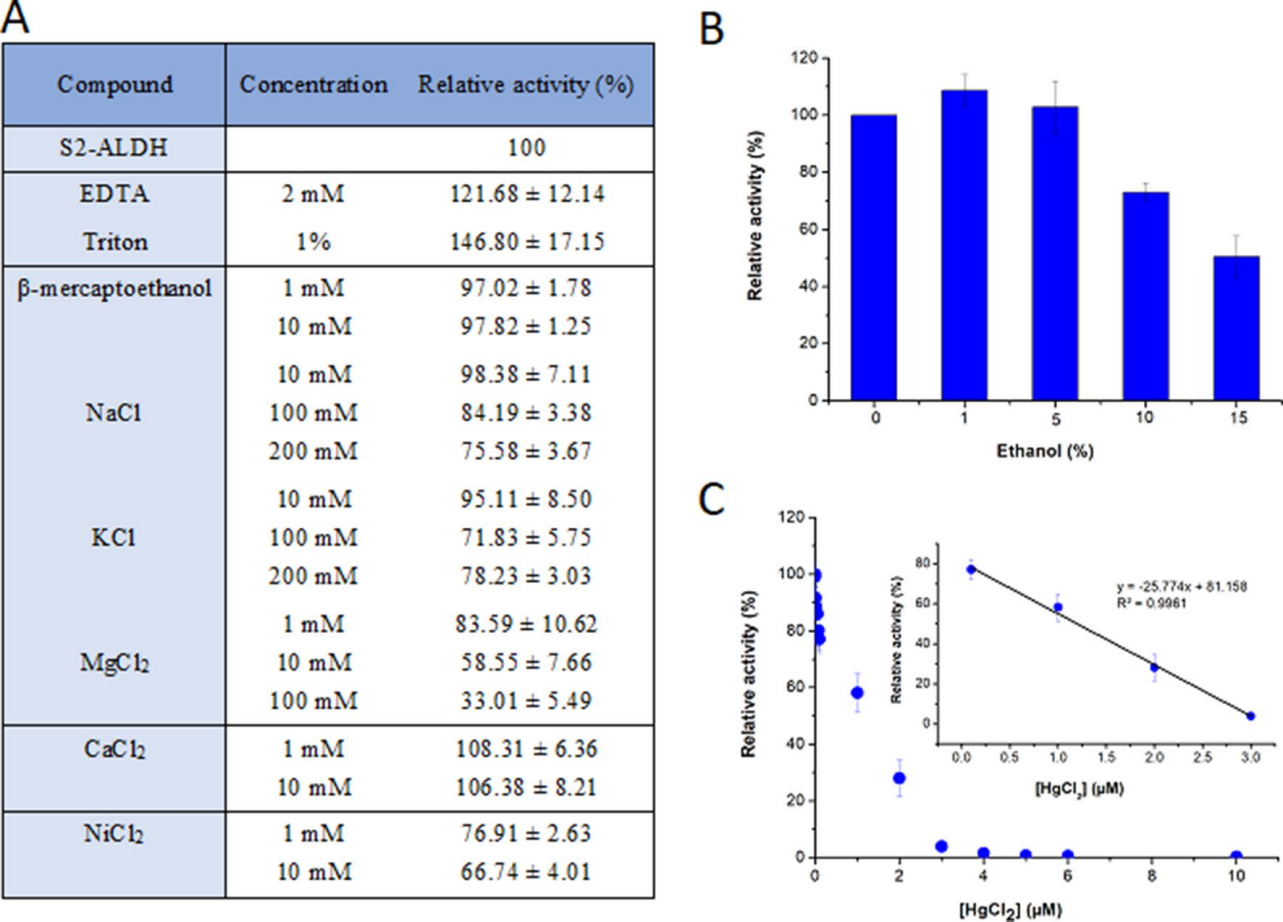
Effect of various compounds on the S2-ALDH activity. The ALDH activity was measured at 25 °C when using 10 mM propionaldehyde and 10 mM NAD^+^ in the presence of salts and compounds (**A**), 0–15% ethanol (**B**), and 0–6 µM HgCl_2_ (**C**). The average and standard deviation values of the relative activity were calculated from triplicate experiments, considering 100% activity in the absence of additives corresponding to 0.68 U/mg. The I50 for HgCl_2_ was calculated from the linear part of the plot (inset panel C).

*Effect of metal ions and additives* on the NAD-dependent activity when using propionaldehyde as substrate showed an overall stable response (Fig. 2). No inhibitory effect was observed in the cases of EDTA (2 mM), Triton (1%) and betamercaptoethanol (1–10 mM), the first two tested compounds inducing even a slight activation of 121.68% and 146.80%, respectively (Fig. 2A). This cold-active ALDH showed no inhibition in the presence of ethanol at concentrations up to 5%, and a residual activity of 73% and 50% in the presence of 10% and 15% EtOH, respectively (Fig. 2B). NaCl and KCl concentrations of 100 mM and 200 mM induced a moderate activity loss up to 26%, while no inhibition was observed by 1 mM of both these monovalent ions (Fig. 2A). Moreover, the cold-active S2-ALDH had a different response to various divalent ions, being inhibited by 41% and 77% in the presence of 10 mM and 100 mM MgCl_2_, respectively, while CaCl_2_ had no impact up to 10 mM. In addition, 1 mM NiCl_2_ inhibited the S2-ALDH enzyme by 23%, with a slight increase (35%) at concentrations of 10 mM (Fig. 2A). Such high resilience to monovalent and divalent ions of this cold-active enzyme was also observed in the case of the hyperthermophilic *S. tokadaii* ALDHs^48^, except for Mg^2+^ that showed no affect. Interestingly, in the case of HgCl_2,_ S2-ALDH exhibited a high sensitivity to this divalent ion, with 15% residual activity in the presence of 10 nM HgCl_2_, and a calculated I50 value of 1.21 µM (Fig. 2C). The total inhibition of both ALDH from the thermophilic *Geobacillus thermodenitrificans* NG80-2^55^ and the cold-active F-ALDH originating from the same bacterial host strain as S2-by 1 mM and 0.5 mM HgCl_2_, respectively, revealed the strong response to this heavy metal of this class of enzymes independent of their origin. Moreover, the high sensitivity to mercury of the novel S2-ALDH could constitute a starting point for develping stable sensors for detecting traces of this heavy metal in various environments.

### Storage, lyophilization and thermal stability of S2-ALDH

**Figure 3:**
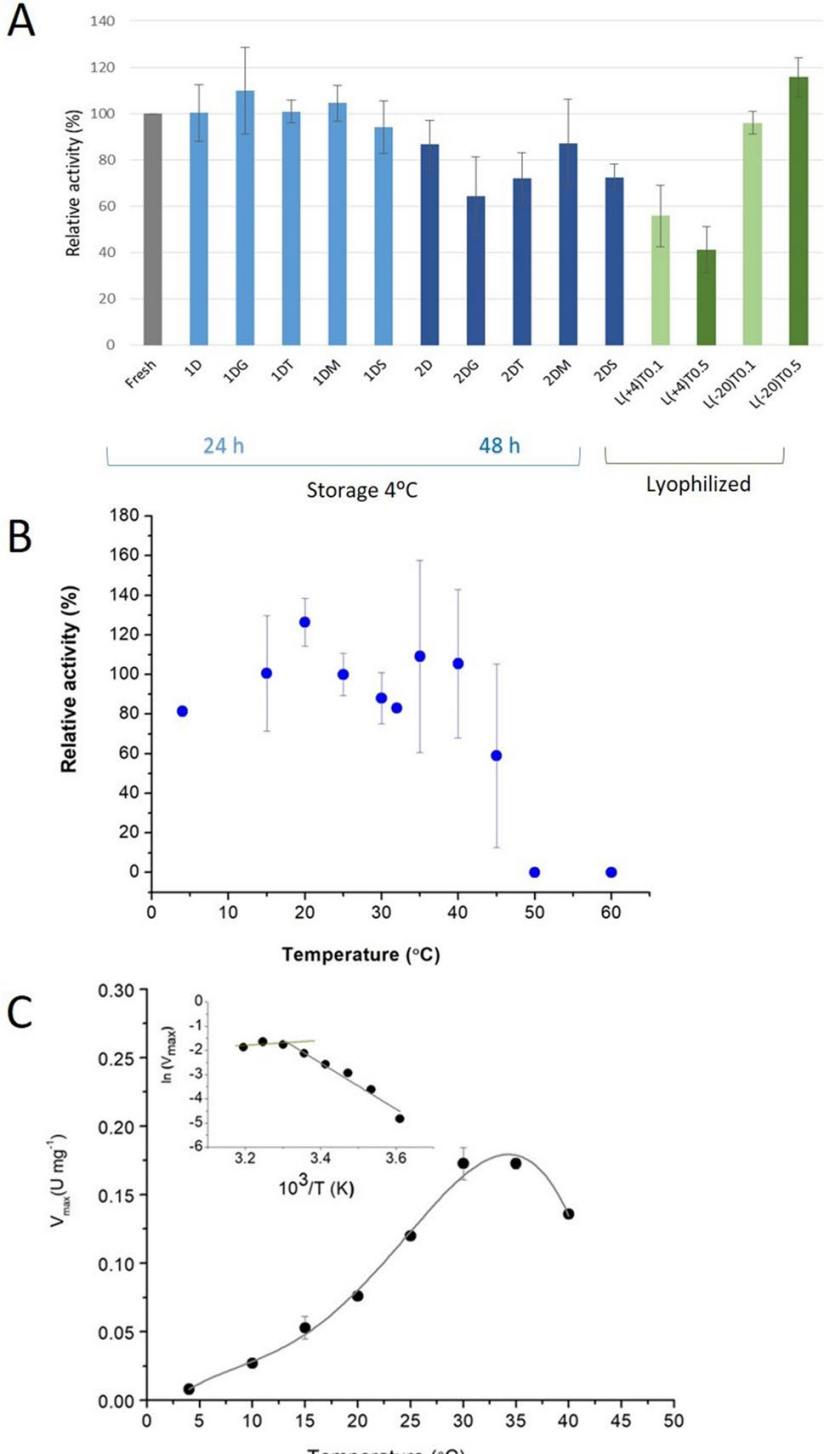
Temperature stability and activity response of S2-ALDH. (**A**) Stability of liquid and lyophilized S2-ALDH. The activity of the fresh S2-ALDH stored at 4 °C (blue) for 1 day (light blue) and 2 days (dark blue) in the absence of stabilizers (1D, 2D), and in the presence of 20% glycerol (1DG, 2DG), 1 M trehalose (1DT, 2DT), 0.5 M mannose (1DM, 2DM), 1 M sucrose (1DS, 2DS), and lyophilized enzyme (green) with 0.1 M trehalose (light green) and 0.5 M trehalose (dark green) after 24 h storage at 4 °C and − 20 °C was measured as described in “Methods”. Relative activity was calculated as percentage of the freshly purified S2-ALDH, where 100% activity corresponded to 0.74 U/mg; (**B**) thermal stability after incubation for 15 min at various temperatures (4–60 °C), and assayed at 25 °C using 10 mM propionaldehyde and 10 mM NAD^+^; (**C**) temperature effect on the activity of S2-ALDH. Propionaldehyde saturation curves were performed at various temperatures (4–40 °C), and the maximum velocity (V_max_) was calculated as described in “Methods”. Corresponding Arrhenius plot (inset).

The stability of the purified S2-ALDH when stored as liquid and lyophlized forms under various conditions, and the thermal stability after exposure to higher temperatures that those of the natural habitat of *Flavobacterium* PL002^42^ were determined (Fig. 3) in order to evaluate the potential of this cold-active enzyme for practical applications in analytical, industrial and biotechnological processes.

Storage of S2-ALDH at 4 °C for 2 days in the presence and absence of different additives showed full preservation of the enzyme activity for 24 h, with a partial activity loss (13–35%) after 48 h without additives, and in the presence of 20% glycerol, 1 M trehalose, 1 M sucrose or 0.5 M mannose, respectively (Fig. 3A). Moreover, the lyophilized enzyme treated with 0.1 M or 0.5 M trehalose was fully stable for 24 h at − 20 °C, but loosed 43–56% activity when stored at 4 °C (Fig. 3A). These data constitute a good starting point for longer stability studies.

Investigation of the thermal stability of the Antarctic ALDH after exposure to different temperatures revealed a fully preserved activity up to 40 °C, followed by an abrupt decrease at above 45 °C and complete inactivation at 50 °C (Fig. 3B).

This high stability of this cold-active enzyme to temperatures above 25 °C commonly used in industrial applications was also observed for other ALDHs originating from Antarctic habitats, such as the recently described F-ALDH from *Flavobacterium* PL002^39^ and the homologous enzyme from the psychrotrophic marine *F. frigidimaris* (*Cytophaga sp*)^41^.

The temperature effect on the S2-ALDH activity was determined by conducting the assays at variable temperatures ranging between 4 and 40 °C, taking into account the thermal stability of the enzyme (Fig. 3C). As expected, the activity increased rapidly with the temperature and started to decrease above 35 °C when the protein began to be affected by thermal denaturation. The Arrhenius representation (Fig. 3C, inset) corresponded to a bi-phasic plot with a calculated activation energy shift from E_a_^10–30 °C^ = 64.77 J/K mol to E_a_^30–35 °C^ = 18.72 J/K mol, suggesting a conformational change that occurred at 30 °C. This temperature effect on enzymes’ tertiary and quaternary structure entrained a wider energy shift and a lower activation energy at low temperature in the case of S2-ALDH as compared to that of the F-ALDH from the same host (from E_a_^10–30 °C^ = 76 J/K mol to E_a_^30–35 °C^ = 19 J/K mol) that occurred at the same temperature threshold (30 °C)^39^. These data suggested similar overall intramolecular interactions involved in catalysis for the two cold-active ALDHs and similar activation energy required at higher temperatures, while a favored catalysis (lower E_a_) at low temperatures in the case of S2-ALDH. Moreover, the homologous enzyme from the psychrophilic *F. frigidimaris*^41^ required a higher activation energy of 27 J/K mol above 30 °C and comparable (57 J/K mol) at low temperatures, as a hint for particular thermal adaptation mechanisms of these psychrotolerant and psychrophilic species.

### Kinetic parameters of recombinant S2-ALDH

In order to determine the apparent affinity for acetaldehyde and the catalytic efficiency of this recombinant cold-active enzyme, saturation curves for the NAD^+^-dependent reaction of acetaldehyde were carried out at 25 °C for both the substrate and cofactor. In this case, the calculated steady state kinetic parameters of the S2-ALDH (Table 2) indicated a k_cat_ value of 0.432 ± 0.025/s and K_m_ of 1.88 ± 0.70 mM for acetaldehyde, corresponding to a catalytic efficiency of 229.8 ± 85.2/M s. Meanwhile, the k_cat_ for NAD+ was of 0.121 ± 0.026/s and K_m_ in the mM range (2.87 ± 0.48 mM), with calculated catalytic efficiency of 42.16 ± 7.05/M s (Table 2).

**Table 2:** Steady state kinetic parameters of S2-ALDH at 25 °C.

Enzyme	Variable substrate	*K*_m_ (mM)	Vmax (µmol/min mg)	*k*_cat_ (/s)	*k*_cat_/*K*_m_ (/M s)
S2-ALDH	Acetaldehyde	1.88 ± 0.70	2.64 ± 0.15	0.432 ± 0.025	229.8 ± 85.2
	NAD^+^	2.87 ± 0.48	0.74 ± 0.16	0.121 ± 0.026	42.16 ± 7.05

In comparison with the homologous enzymes from *Saccharomyces cerevisiae var. boulardii*, NCYC 3264, the catalytic efficiency of S2-ALDH for the utilization of acetaldehyde at 25 °C was two-fold (14.3/M s) and three-fold (7.38/M s) higher, respectively, than that of the mitochondrial (ALD4) and cytosolic (ALD6) ALDHs from yeast measured at 30 °C^56^, corresponding to a more potent catalyst al low temperatures than these mesophilic enzymes. Moreover, the ALDH from the hyperthermophilic *S. tokadaii* strain 7 had a threefold higher Km (6.38 mM) when oxidizing this substrate at 80 °C, and a slightly lower catalytic efficiency (180/M s) than that of the cold-active counterpart, with a severe activity reduction (by 90%) at temperatures < 30 °C relative to the optimal of 80 °C for this enzyme^48^.

In this context, the high stability of the investigated S2-ALDH within a temperature range suitable for industrial and biosensing applications, and stability after lyophilization, as well as the high activity and catalytic efficiency at 25 °C constituted cumulative favorable conditions for developing applicative model systems using this Antarctic enzyme for acetaldehyde monitoring in liquid phase. In particular, the preference for acetaldehyde as substrate, combined with the high ratio between the typical levels of acetaldehyde and other aldehydes in wine and the lack of sensitivity towards ethanol, recommended the recombinant cold-active S2-ALDH as an advantageous catalyst for acetaldehyde determination in wine.

### Application of S2-ALDH in electrochemical assays for acetaldehyde determination in wine

In order to test the applicative potential of S2-ALDH as biocatalyst, an electrochemical assay for the determination of acetaldehyde in wines was developped and optimized. This application was based on measuring the concentration of NADH formed in the S2-ALDH enzymatic reaction which was oxidized on CNT electrodes, the intensity of the generated anodic current being proportional to the concentration of acetaldehyde in the sample. Carbon nanotubes electrodes were selected taking advantage of their antifouling surface, electrocatalytic characteristics for the detection of NADH, and high sensitivity when implemented as electrochemical transducers in biosensors^57^.

The preliminary characterization of the CNT electrodes by Raman, SEM and cyclic voltammetry (Supplementary Fig. 5S) confirmed the advantages of these electrodes for electrochemical detection, considering the high surface to volume ratio (specific surface BET of 300 m^2^/g), favorable morphology of the CNT layer for sensitive electrochemical detection, and the electrocatalytic effect for NADH oxidation. When used in chronoamperometry measurements at 0.5 V, these electrodes responded linearly to NADH concentrations in the range of 12.5–250 µM (R^2^ = 0.9992), with a sensitivity of 1.15 ± 0.16 μA L/mmol and a detection limit of 10 µM (Supplementary Table 2S). These features were similar to those reported for other sensors, and appropriate for substrates detection of NAD^+^-dependent dehydrogenases^40,58^.

**Figure 4:**
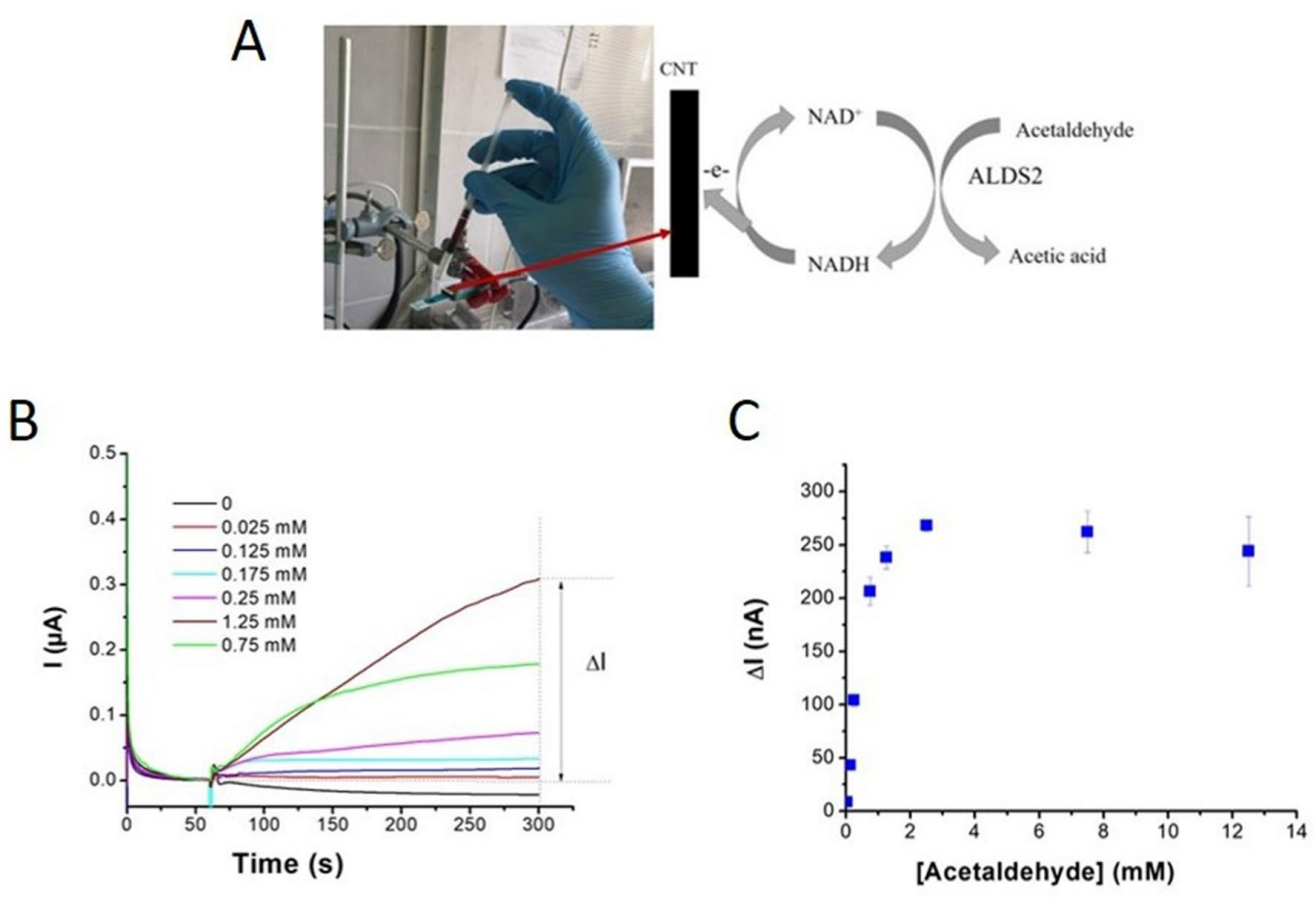
Determination of acetaldehyde using S2-ALDH and CNT electrodes. (**A**) Experimental setup; (**B**) intensity versus time plots for different concentrations of acetaldehyde tested by the assay based on S2-ALDH; (**C**) the variation of the current intensity between 300 and 60 s with the concentration of acetaldehyde for the measurement of the apparent Km.

These pre-requisits for acetaldehyde concentration measurement using S2-ALDH were verified by a typical detection scheme^40^ which minimized the reagents amount used for the assay (Fig. 4A).

Based on the current intensity increase (ΔI) with the concentration of acetaldehyde (Fig. 4B), an apparent Km_app_ of 1.10 ± 0.52 mM was calculated for S2-ALDH (Fig. 4C). Five calibrations were performed in the range of 0.025 mM 0.75 mM acetaldehyde over a 5-week period, with on average of 24 tests in the same day, using one sensor per calibration. (The repeatability, evaluated based on the average RSD (n = 3 replicates) of responses measured with the same electrode for various concentrations of acetaldehyde within the linear range was 11.3%, varying in the 1.2–35.1% interval. The average assay sensitivity was 327.1 ± 75.0 nA L/µmol, and the calculated detection limit was 17 µM acetaldehyde based on the threefold standard deviation of the blank divided by the slope of the calibration curve. These characteristics indicated appropriate ruggedness of the assay and suitability for the measurement of acetaldehyde in wine, taking into considertion the average acetaldehyde content in this matrix of up to 4.8 mM^4^.

### Optimization of wine pre-treatment for the electrochemical assays based on S2-ALDH

Analysis of acetaldehyde concentration in wines was carried out after optimizing the experimental conditions and wine pre-treatment, taking into consideration the pH of various wines ranging in the 3.3–3.6 interval, the ethanol content of 11–13%, and the presence of easily oxidizable species including high amounts of phenolics. Consequently, all acetaldehyde solutions used for calibration were prepared in model wine solution to avoid any bias on the activity measurements of S2-ALDH.

As previously shown (Fig. 2B), this cold-active ALDH preserved full activity in the presence of 5% ethanol. Nonetheless, the presence of phenolic compounds oxidized at the relatively high potential (0.5 V) was likely to induce electrochemical interferences. Therefore, to eliminate interferences^59^, the wine was filtered through a cartridge filled with PVPP and activated charcoal using an adaptation of a literature procedure^10^.

**Figure 5:**
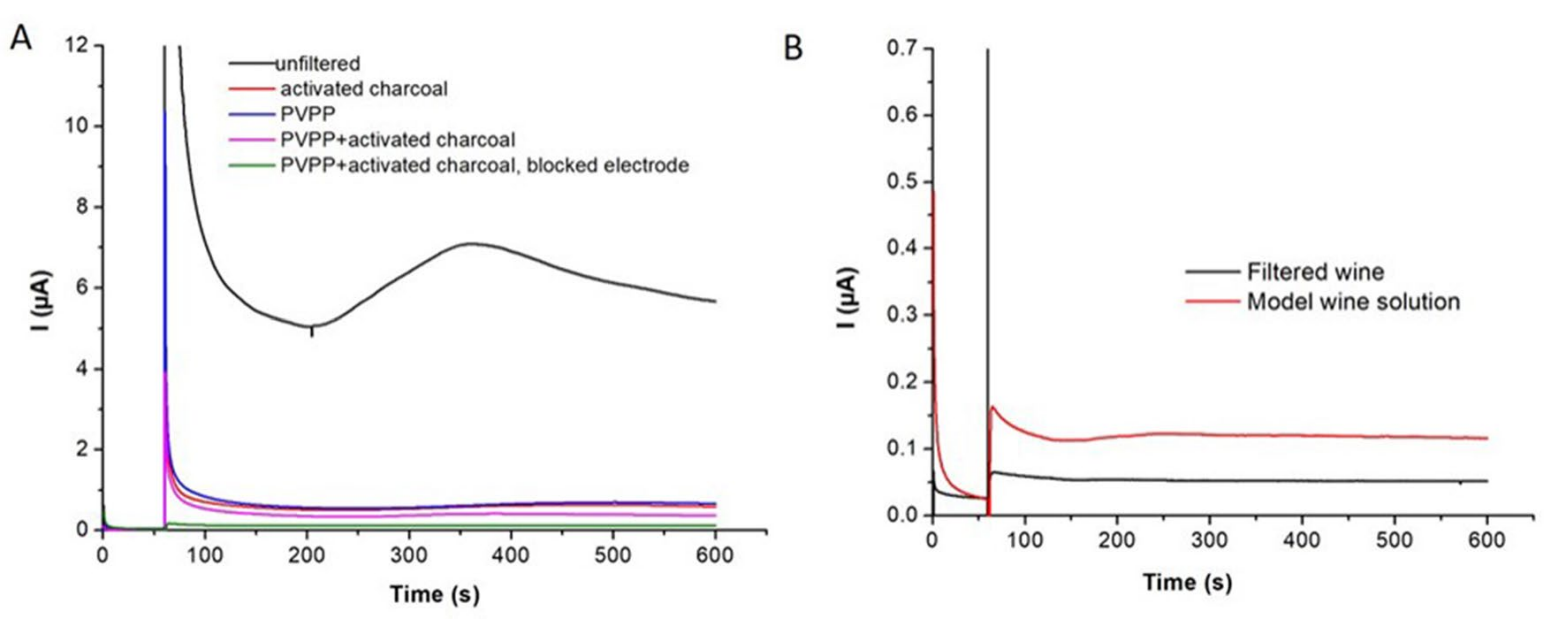
Optimization of wine pre-treatment. (**A**) Chronoamperograms of wine samples unfiltered or filtered through PVPP, activated charcoal or a combination of both, analyzed with a CNT electrode polarized at + 0.5 V; (**B)** comparison of the anodic current recorded for a red wine filtered through a cartridge filled with PVPP and activated charcoal and for a model wine solution, analyzed with a CNT electrode “blocked” with BSA polarized at 0.5 V.

The sample treatment efficiency was evaluated at 0.5 V using CNT-electrodes (Fig. 5). Among variable conditions tested during optimization (reported in Supplementary information), the smallest anodic current at 0.5 V, potentially interfering with the analysis of acetaldehyde, was obtained for the wine filtered through a cartridge filled with PVPP and activated charcoal, and analyzed on a CNT electrode “blocked” with BSA (Fig. 5A). The current intensity due to the oxidation of phenolic compounds from a red wine decreased by ~ 98% from 5.69 to 0.12 µA after filtration through the PVPP/activated charcoal cartridge (Fig. 5A). The remaining small anodic current was still significantly higher than that recorded for a model wine solution under the same conditions, and was stable after 100 s, indicating the fast oxidation of the residual phenolics in wine. The difference between the current intensity at 400 s and 100 s (7.6 ± 0.07 nA) was similar for the model wine solution and the filtered red wine (Fig. 5B). Consequently, the current intensity at 100 s was considered as baseline, and the variation between 100 and 400 s (mainly due to NADH formed in the S2-ALDH-catalyzed reaction) corresponded to the analytical signal.

For acetaldehyde measurements, the calibration was performed at 17 °C in order to mimic the typical temperatures in cellars and avoid evaporation of acetaldehyde (Supplementary Fig. 6SA). In this case, the sensitivity of the test was of 508.4 µA L/mmol. The determined linear range of the assay (R^2^ = 0.9927) was of 0.125–2.5 mM acetaldehyde in the electrochemical cell (Supplementary Fig. 6SB). Considering the dilution, this corresponds to 0.5–10 mM (4.4–440 mg/L) in wines. These corroborated data demonstrated that the assay was appropriate for testing wines, considering their average content of 34–211 mg/L acetaldehyde^4^.

The accuracy of the electrochemical assay of acetaldehyde in wines using this method was demonstrated in comparison with a reference method that relies on a commercial kit using an aldehyde dehydrogenase from yeast^10^ and spectrophotometric detection (Table 3).

**Table 3:** Analysis of acetaldehyde concentration in wines using alternative methods.

Wine sample	Acetaldehyde concentration (mg/L)	
	SP Commercial kit	EL S2-ALDH
Red house wine	345.4 ± 48.0	431.7 ± 50.2
White wine no 6	375.3 ± 109.0	394.5 ± 7.4
Red wine CF3000	453.7 ± 145.5	434.3 ± 7.4
Rose house wine	30.7 ± 3.7	28.1 ± 0.8
White wine no 2	20.2 ± 1.4	22.2 ± 6.0
Red wine I 46	4.8 ± 0.2	37.3 ± 0.3
Red wine Ferm	559.6 ± 152.0	529.6 ± 98.0
Red wine Negru Aromat	15.8 ± 2.9	52.3 ± 5.8

(SP), spectrophotometric method with commercial enzymatic kit; (EL), electrochemical assay in liquid phase at 17 °C using S2-ALDH.

In the case of all eigtht analyzed wine samples (2 white, 1 rosé and 5 red wines), the acetaldehyde concentration measured by the two methods was in good agreement (Table 3), with a correlation coefficient of R^2^ = 0.9747 and a slope of 1.0183 (Supplementary Fig. 6SC). As compared to the spectrophotometric test, the proposed electrochemical assay is simpler, faster, uses cost-effective and portable equipment, and can be easily adapted for in situ measurements in wine cellars.

These corroborated data confirmed the utilization of the novel cold-active ALDH as an effective biocatalyst for monitoring acetaldehyde in wine, with extensive applicative potential in biosensing. To further explore this potential, immobilization of S2-ALDH will be conducted based on crosslinking with glutaraldehyde, as demonstrated for the homologous enzyme originating from the same bacterial species^39^ and Ni-histidine affinity for developing enzymatic biosensors for detection of aldehydes in alcoholic beverages and other relevant industrial applications.

## Conclusions

A new cold-active bacterial aldehyde dehydrogenase was obtained originating from the Antarctic marine strain *Flavobacterium* PL002 by cloning and expression in *E. coli*. This recombinant S2-ALDH enzyme exhibited a broad substrate specificity encompassing aliphatic and aromatic aldehydes and particular functional characteristics as compared with ALDHs from other sources. In addition to the high sensitivity to mercury ions with I50 in the µM range suggesting that S2-ALDH was a good candidate for the detection of this heavy metal, the functional profile of the novel cold-active ALDH revealed compelling advantages for practical applications in biotechnology and biosensing. These features corroborate a high stability to storage and lyophilization, neutral optimum pH, high substrate specificity for acetaldehyde (a particular asset for wine analysis), high tolerance for a series of compounds including ethanol, and relatively high catalytic efficiency at low temperatures. The neutral optimum pH is in particular advantageous for mediator-based electrochemical sensors and coupled chemical and enzymatic reactions (e.g. for cascade reactions in biocatalysis).

The different substrate specificity and catalytic efficiency of the NAD^+^-dependent acetaldehyde oxidation as compared to both the commercial enzyme from yeast and the previously characterized cold-active F-ALDH open the way for using this cold-active ALDH as component of bioelectronic tongues for the specific detection of aldehydes.

The applicative potential of the newly characterized cold-active microbial enzyme S2-ALDH as potent biocatalyst for industrial processes and biosensing was evidenced by the enzymatic electrochemical detection of acetaldehydes in wines. The assay presented here is simple to perform, and the costs were decreased by the use of low fouling carbon nanotube electrodes, that are used for multiple tests.

These corroborated data support the use of S2-ALDH as valuable catalyst in developing easy to use kits for acetaldehyde measurements in wine. Alternatively, biosensors with the immobilized recombinant Antarctic enzyme could be envisaged for various applications targetting other relevant aldehydes. Experiments for obtaining stable enzymatic inks based on S2-ALDH for developing printed, highly sensitive detection interfaces are under way in our laboratory, demonstrating the applicative potential of this Antarctic enzyme.

## Methods

### Reagents and materials

Polyvinylpolypyrollidone (PVPP) was from Supelco, Switzerland. Activated charcoal, tartaric acid, sodium phosphate, monobasic and dibasic were from Sigma Aldrich (Merck), Germany. Nicotinamide adenin dinucleotide (NAD^+^) and isopropyl-β-d-thiogalactoside (IPTG) were from Carl Roth, Germany. NADH and the spectrophotometric kit for acetaldehyde were from Roche (Merck), Germany. Screen printed electrodes modified with carbon nanotubes (DRP 110 CNT) and polyamide membranes covering the screen-printed 3-electrode system for the analysis of small volumes of sample (7.5–15 μL) were from Metrohm Dropsens, Oviedo, Spain. Ethanol was from Chimreactiv S.R.L. (Bucharest, Romania). The enzymatic UV spectrometric kit for acetaldehyde was from R-Biopharm, Darmstadt, Germany. The aldehydes were from Acros Organics, Geel, Belgium.

A model wine solution (5 g/L tartaric acid, 13% ethanol, pH 3.6^60^ was used for preparing the standard acetaldehyde solutions and for diluting the samples for the wine analysis. All acetaldehyde solutions were kept at 4 °C before use to avoid evaporation. The wines tested included two house wines without added preservatives (one rosé, one red) and six commercial wines, kindly provided by the Research and Development Institute for Vine and Wine, Valea Calugareasca, Romania.

### Cloning and heterologous expression of *Flavobacterium* PL002 *S2-ALDH* gene

The *S2-ALDH* (1365 bp) gene (Supplementary Fig. S1) identified in the *Flavobacterium* PL002 genome sequence^42^ was synthesized (ATG Biosynthetics GmbH, Merzhausen, Germany) and cloned into the pHAT2 His-tag expression vector (EMBL, Heidelberg, Germany) using the *Nco*I/*Bam*HI restriction sites. Gene expression of the resulting pS2-ALDH recombinant plasmid was performed in *Escherichia coli* BL21(DE3) (Thermo Fisher Scientific, Massachusetts, USA) after induction for 6 h at 25 °C in the presence of 1 mM IPTG. The induced cells were separated by centrifugation at 9000 × *g* for 10 min (4 °C) and stored at − 80 °C.

### Purification of the recombinant S2-ALDH

The recombinant enzyme S2-ALDH appending an N-terminal His-tag polypeptide to the psychrophilic protein was purified by affinity chromatography using Ni–NTA agarose (Qiagen, Hilden, Germany)^61^. After induction (200 mL culture), the cells were resuspended in 6 mL of buffer A (100 mM TrisHCl pH 8, 200 mM NaCl) and dissrupted by 5 min ultrasonication cycles alternating 5 s pulses and 60 s pauses, using a Sonopuls ultrasonic homogenizer (Bandelin, Berlin, Germany). Following the centrifugation of the extract at 16.000 × *g* for 30 min at 4 °C, the soluble fraction was passed on a 1-mL Ni–NTA agarose (Qiagen, Hilden, Germany) column equilibrated with buffer A and washed with 10 mL buffer A and 3 mL buffer A containing 30 mM imidazole. The recombinant S2-ALDH was further eluted in the presence of 50–100 mM imidazole. The protein fractions were examined by SDS-PAGE and desalted using 7 K MWCO Zeba Spin Desalting columns (ThermoFisher Scientific, Massachusetts, USA). The resulted enzyme fractions were store at – 20 °C in 100 mM TrisHCl pH 8 buffer containing 20% glycerol.

### Aldehyde dehydrogenase assays

The activity of free S2-ALDH was measured spectrophotometrically in a FLUOstarOmega microplate reader (BMG Labtech, Offenburg, Germany) by monitoring the rate of NADH formation at OD_340 nm_^62^. The reaction was measured at 25 °C using a FLUOstarOmega microplate reader (BMG Labtech, Offenburg, Germany), in the presence of 10 mM aldehyde and 10 mM NAD^+^, using 100 mM potassium phosphate buffer pH 7.5, and initiated with 6 µg S2-ALDH. One unit of ALDH was defined as the amount of enzyme producing 1 μmol NADH per minute, using the molar absorption coefficient ε_NADH_ = 6.22 × 10^3^ L/mol cm.

The optimal pH was determined in the presence of various buffers encompassing 100 mM potassium phosphate buffer (pH 6.0–7.5), Tris buffer (pH 7.5–9), and glycine-KOH buffer (pH 9.0–10.5).

Substrate specificity was determined for a series of aliphatic (acetaldehyde, propionaldehyde, valeraldehyde, isovaleraldehyde, butyraldehyde, and hexanal) and aromatic (benzaldehyde, 2-fluorobenzaldehyde) aldehydes at both 1 mM and 10 mM, in the presence of 10 mM NAD^+^.

The effect of 2 mM EDTA, 1% Triton, 1–10 mM betamercaptoethanol, 1–15% ethanol and 1–200 mM metal ions (Na+, K+, Ca2+, Mg2+, Ni2+, Hg2+ on the activity of S2-ALDH was measured at 25 °C in the presence of varrious concentrations of salts and compounds, using 10 mM propionaldehyde and 10 mM NAD^+^.

The thermal stability was determined by incubating the enzyme for 15 min at various temperatures in the 4–50 °C interval, and further measuring their residual activity at 25 °C. The effect of temperature on the reaction rate was obtained by measuring the activity at different temperatures ranging from 4 to 40 °C, in the presence of 10 mM propionaldehyde and 10 mM NAD^+^. Arrhenius plot was used for calculating the activation energy of the S2-ALDH catalyzed reaction (Atkins and De Paula, 2006).

S2-ALDH stability was determined after incubation at 4 °C for 24 h and 48 h in the presence and absence of 20% glycerol, 1 M trehalose, 0.5 M mannose, or 1 M sucrose. Lyophilization of S2-ALDH samples (100 µL) using a Martin Christ Alpha 1–4 LO plus Freeze Dryer Lyophilizer was performed in the presence and absence of after adding 0.1 M trehalose and 0.5 M trehalose by freeze-dry for 3 h after incubation of the freshy purified enzyme at − 20 °C for 16 h. Stability of the lyophilized enzyme was monitored after storage at 4 °C and − 20 °C for 24 h, respectively, by measuring the ALDH activity prior and post treatment under standard conditions. The residual activity expressed as percentage relative to the ALDH activity of the untreated enzyme was measured under standard conditions.

The saturation curves were determined at 25 °C by alternatively varying the substrate acetaldehyde and cofactor NAD^+^ concentrations, while keeping saturating (10 mM) the alternative substrate, and fit to the Michaelis–Menten equation for calculating the K_M_ and V_max_ kinetic parameters.

### Sequence analyses

Primary structure analysis of S2-ALDH providing the theoretical molecular weight (MW), isoelectric point (pI), and aminoacid composition was performed using ExPASy ProtParam platform (https://web.expasy.org/cgi-bin/protparam/protparam)^63^. Sequence similarity and identity percentages between the Antarctic enzyme and homologous ALDHs were calculated using Emboss Needle pair alignment tool (http://www.ebi.ac.uk/Tools/psa/emboss_needle/)^64^. Multiple sequence alignment of ALDHs primary structures was carried out using the CLUSTAL OMEGA EMBL-EBI (1.2.4) software^64^.

Alignment and phylogenetic analysis of ALDH sequences were performed using the MAFFT online service version 7^65,66^. Phylogeny was carried out using 15 amino acid sequences with a total of 454 positions after elimination of all positions containing gaps and missing data. Initial trees for the heuristic search were obtained by applying Neighbor-Join algorithm to a matrix of pairwise distances using a JTT model. The tree topography was evaluated using the bootstrap analysis of 1000 repetitions. The Itol online service (https://itol.embl.de/) was used to visualize and edit the phylogenetic tree^67^.

### Analysis of wine samples by the spectrophotometric aldehyde dehydrogenase test

The wines and the standard acetaldehyde solutions were pretreated immediately before the analysis by filtration through a 1 mL cartridge containing 0.15 g PVPP and 0.025 g activated charcoal (the optimization of wine pre-treatment is reported in Supplementary Fig. 6S). A commercial kit from Roche (Germany) was used for evaluating the acetaldehyde quantity by spectrophotometry using the yeast ALDH. Spectrophotometric measurements at 340 nm were performed at room temperature with a UV–VIS Evolution 600 spectrophotometer (Thermo Scientific, Loughborough, United Kingdom) equipped with VISION PRO software.

### Electrochemical assays

A VSP potentiostat (BioLogic, France) equipped with the EC Lab software was used for most electrochemical tests. A PalmSens4 potentiostat (PalmSens, Netherlands) equipped with the PSTrace software was also used for the amperometric tests at 17 °C. CNT electrodes (catalog number DRP 110CNT, Metrohm Dropsens, Spain) consist in 3 coplanar electrodes printed on a ceramic support, with a 4 mm diameter working electrode made of carbon modified with multi-walled carbon nanotubes, a carbon counter electrode and a silver reference electrode. Carbon electrodes (DRP110, Metrohm Dropsens, Spain) with similar characteristics but with the working electrode made of bare carbon were also used in some tests. All measurements were performed in triplicate. The temperature and humidity during the electrochemical assays of acetaldehyde in wines were monitored by a sensor placed in the vicinity of the electrode. Amperometric assays were performed at + 0.5 V with the screen-printed Ag electrode used as reference. The electrodes were fixed horizontally and the sample was added at 60 s after applying the potential. The analytical signal consisted in the difference between the current intensity at two different times, t_1_ and t_0_, which were optimized depending on the test.

For the *c*hronoamperometric measurements of NADH, 75 µL buffer 0.2 M phosphate buffer pH 7.5 were mixed with 25 µL NADH aqueous solutions of concentrations 2.5–250 µM. The difference between the current intensity at 180 s and 60 s was correlated with the NADH concentration.

For calculating the Km for acetaldehyde, the final concentrations in the electrochemical cell were 7.5 mM NAD^+^, 0.2 U/mL S2-ALDH and 0.025–12.5 mM acetaldehyde. The difference in current intensity between 300 and 60 s was correlated with the concentration of acetaldehyde in the sample.

For the enzymatic assay of acetaldehyde in wines at 17 °C, 75 µL of S2-ALDH (0.26U/mL) and NAD^+^ (10 mM) in 0.2 M phosphate buffer pH 7.5 were mixed with 25 µL solution of acetaldehyde of different concentrations in the range 0.1–10 mM prepared in model wine solution. The difference in current intensity between 400 and 100 s was taken as the analytical signal.

### Raman analysis

Raman measurements were carried out with SPELEC RAMAN (Metrohm DropSens, Spain), a compact instrument with a laser source of 785 nm. This instrument was connected to a bifurcated reflection probe (DRP-RAMANPROBE, Metrohm DropSens) and a specific cell for screen-printed electrodes SPEs (DRP-RAMANCELL, Metrohm DropSens) was used. The SPELEC RAMAN instrument was controlled by DropView SPELEC software. Integration time for raman spectra was 20 s.

### Scanning electron microscopy

Images of the electrode surfaces were obtained with a JEOL JSM-6100 scanning electron microscope (20 kV, Japan), after the sputtering of 20 nm gold layers over the samples (Gold sputter coater Balzers SCD004, Liechtenstein).

## Supplementary Information

Supplementary Information.

## Data Availability

All data supporting the conclusions of this article are included in the manuscript. The annotated genome sequence of *Flavobacterium* PL002 was deposited in DDBJ/ENA/GenBank under the accession number MQTN00000000.1. The corresponding aminoacid sequence of S2-ALDH used in the current study has the NCBI Reference Sequence number WP_173857136.1.
