# Supplementary material for: Antarctic aldehyde dehydrogenase from Flavobacterium PL002 as a potent catalyst for acetaldehyde determination in wine
Source: Sci Rep. 2022 Oct 15;12:17301. doi: 10.1038/s41598-022-22289-8 (PMC9569350; doi:10.1038/s41598-022-22289-8)
Supplement: Supplementary file 1 — Supplementary Information. [file 41598_2022_22289_MOESM1_ESM.pdf]

SUPPLEMENTARY MATERIAL

Antarctic aldehyde dehydrogenase from *Flavobacterium* PL002 as a potent catalyst for acetaldehyde determination in wine

Paun VI [1#], Banciu RM [2#], Lavin P[3], Vasilescu A[2], Fanjul-Bolado P[4], Purcarea C[1*]

1 Department of Microbiology, Institute of Biology, 296 Splaiul Independentei, 060031 Bucharest, Romania

[2] International Centre of Biodynamics, 1B Intrarea Portocalelor, 060101 Bucharest, Romania

[3] Departamento de Biotecnología, Facultad de Ciencias del Mar y Recursos Biológicos, Universidad de Antofagasta, Antofagasta 1240000, Chile

[4] Metrohm DropSens, S.L.,Vivero de Ciencias de la Salud,C/Colegio Santo Domingo de Guzmán s/n, 33010 Oviedo (Asturias), Spain

Correspondence: cristina.purcarea@ibiol.ro

# authors with equal contribution

***ald*S2**

atgtctattcaaacaacaaaccccgctacaaataaagtcataaaatcatttgatgaaatgactgacaagcaagttgaagatgctattgcactttcaa
atactacttttaaagactggaaaaaaaccagctataaagatcgtgctacgatacttcataaagtagcaaaattaatgcgcgctaaaaaaacagaa
cttgccacactaattactttagaaatgggtaaagtttttgctcaatcagaaggggaaattgatcttagtgcaagcatattagattattacgctgacaat
gccgaaaccttttggctgataaaaaattaagccctgaacaaggagaagcttttgtaagaagcagtccaattggagtattatttggcgttatgccat
ggaattttcctttctaccaagtagtacgttttgctgctcctaatataatggtaggaaatactattttattaaaacatgcttcaattgttccgcagtgtgca
attgcaatagacgagttatttaaagaagcaggtgcaccagaaggcttgtacactaatttaatgatctcgggtaaaagaagtaccgcattagttgaa
gatgtaagaatcaagggaatatccttaacaggtagtgaaggtgctggtgctagtatggccgaagctgctggtaaaaaccttaaacgttccgttct
agaattaggaggaagtgatgcttttatcatcttagccgatgcagacattgataaaacagttgatttagcaatttttggaaggatgaataacaacggt
caaagttgtgtagcttcaaaaagatttattgcagttgaagctatagctgatgaatttttagaaaaatttactgctaaagtagctgctttaaaagtagga
gacccaatggatccaagtactgatgttggtcctttgagcagtgaagaagcattacaaggtttattagagcaagtgaaaaaattcgaagctgctgg
agcaaccgtagttgtgggtggaaaaagagcatctaaagaaggggcttatatgcaacctactttattgactaatttaaaaagaggcgaaccgactt
tctacgaagaattatttggacctgtagcatctttttataaagttaaagatgagcaggaagcaattgatttagctaatgactctccctttggattgggag
gatcaatctatactaaagatattaaaaatgccattaaaattgctgatcaaatagactcaggaatggtatttattaactccccctacttggacacaagct
gatttaccatttggaggaactaaacgttctggttacggtcgtgaattgtctgaacaaggaattcaagagtttgtaaataaaaaaattgattagagttag
ttaa

S2-ALDH [WP_173857136]

MSIQTTNPATNKVIKSFDEMTDKQVEDAIALSNTTFKDWKKTSYKDRATILHKVAKLMR
AKKTELATLITLEMGKVFAQSEGEIDLSASILDYYADNAETFLADKKLSPEQGEAFVRSSPI
GVLFGVMPWNFPFYQVVRFAAPNIMVGNTILLKHASIVPQCAIAIDELFKEAGAPEGLYTN
LMISGKRSTALVEDVRIKGISLTGSEGAGASMAEAAGKNLKRSVLELGGSDAFIILADADI
DKTVDLAIFGRMNNNGQSCVASKRFIAVEAIADEFLEKFTAKVAALKVGDPMDPSTDVGP
LSSEEALQGLLEQVKKFEAAGATVVVGGKRASKEGAYMQPTLLTNLKRGEPTFYEELFGP
VASFYKVKDEQEAIDLANDSPFGLGGSIYTKDIKNAIKIADQIDSGMVFINSPTWTQADLPF
GGTKRSGYGRELSEQGIQEFVNKKLIRVS

Figure 1S. *Flavobacterium* PL002 *aldS2* gene nucleotide sequence and corresponding primary structure of S2-ALDH aldehyde dehydrogenase

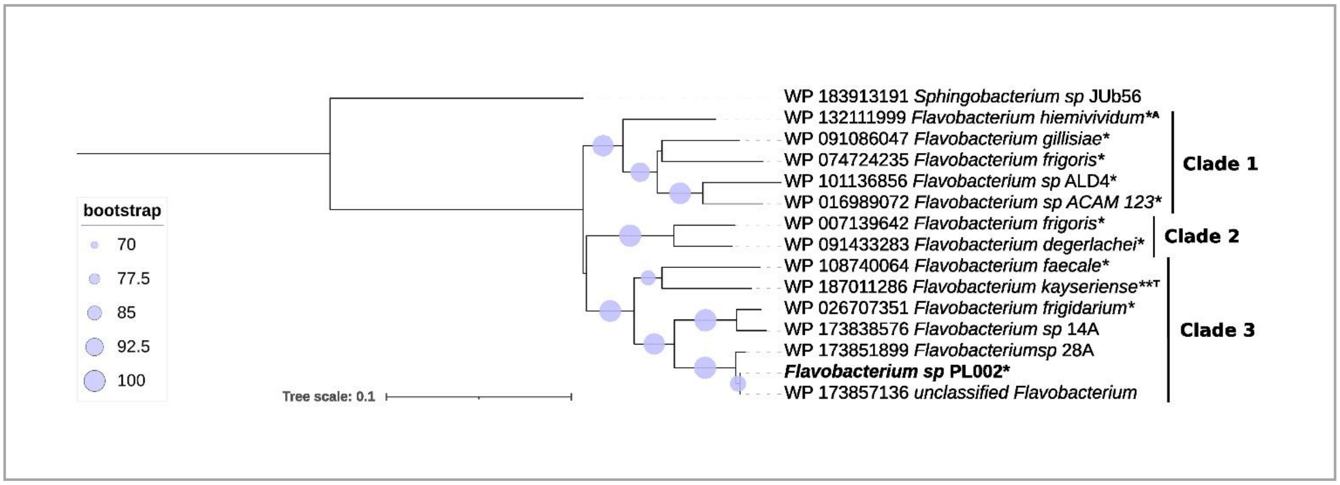

Figure 2S. Neighbour-joining phylogenetic tree of S2-ALDH primary structure. The amino acid sequence of S2-ALDH from *Flavobacterium* PL002 (bold) was used for the phylogenetic tree construction and other *Flavobacterium* species with indicated accession numbers, using the mesophilic *Sfingobacterium* sp. JU56 [WP 183913191] protein sequence as outgroup. All Flavobacterium spp. originated from Antarctica except for *F. kayseriense* from Turkey (T) and *F. hiemivividium* from Arctic (A). Psychrophiles (*); Psychrotolerant (**);

Table 1S. Aminoacid composition of ALDHs

ALDH origin	Number of Cys	Number of Pro	Number of negatively charged residues (Asp + Glu)	Number of positively charged residues (Arg + Lys)	Ratio Arg/(Arg + Lys)
S2-ALDH	2	17	59	52	0.2692
F. frigidarium	4	15	61	53	0.2830
E. coli	7	19	53	46	0.5870
S. aureus	3	18	75	53	0.3396
T. thermophilus	2	27	69	61	0.6557
S. tokadaii str. 7	1	22	62	62	0.4322

```
S2-ALDH     ---------------------------------------MSIQTTNPATNKVIKSFDE   19
FF          ----------------------------------------MENTIIINPATGEKVAEYDR   20
EC          ------------------------------------MTITPATHAISINPATGEQLSVLPW   25
SA          ------------------MELLKHLSQRQYIDGEWVESANKNTRDIINPYNQEVIFTVSE   42
TT          MRYADRVAGISWETIEEVRRRLKERPALHFIAGEFVPSESGETFPSLDPATNEVLGVAAR   60
ST          --------------------------------------MSEVIEIKSPSNLKVIGTVKR   21
                                                        .* . : :

S2-ALDH     MTDKQVEDAIALSNTTF--KDWKKTSYKDRATILHKVAKLMRAKKTELATLITLEMGKVF   77
FF          ITINEAKEKISQANKTY--TGWKLKSFKERSAFMHKLADILDENKEEYAQLATQEMGKVI   78
EC          AGVDDIENALQLAAAGF--RDWRETNIDYRAEKLRDIGKALRARSEEMAQMITREMGKPI   83
SA          GTKEDAERAILAARRAFESGEWSQETAETRGKKVRAIADKIKEHREALARLETLDTGKTL   102
TT          GGEREVDRAAKAAHEAF--QRWSRTKAKERKRYLLRIAELIEKHADELAVMECLDAGQVL   118
ST          MSKDEVRGEIEEAYKGF--ETISRMPLYKRTAILRKVSEILEREQERLARLLAMEAGKPI   79
                :       :   :            *   :  :.. :  .     * :   : *: :

S2-ALDH     AQSEGEIDLSASILDYYADNAETFLADKKLS---------PEQGEAFVRSSPIGVLFGVM   128
FF          GQSRKEIEKCALVCRYYADNAEALLTDEIVK---------TEATKSYVTFQPIGVVLAVM   129
EC          NQARAEVAKSANLCDWYAEHGPAMLKAEPTL---------VENQQAVIEYRPLGTILAIM   134
SA          EESYADMDDIHNVFMYFAGLADKDGGEMIDSP--------IPDTESKIVKEPVGVVTQIT   154
TT          RIVRAQVARAAENFAFYAEYAEHAMEDRTFPV--------DRDWLYYTVRVPAGPVGIIT   170
ST          KDSRVEVMRASRLFRQAAEEAAIVLEGKNYRVDAYEYPPGNENRIVISTREPIGVVTAIL   139
                ::          *   .                                 * *  :   :

S2-ALDH     PWNFPFYQVVRFAAPNIMVGNTILLKHASIVPQCAIAIDELFKEAGAPEGLYTNLMI-SG   187
FF          PWNFPFYQVIRFAAPALMAGNTAVLKHASNVQGCALALEQAFEKAGFPEGAFSNLNI-DS   188
EC          PWNFPLWQVMRGAVPIILAGNGYLLKHAPNVMGCAQLIAQVFKDAGFPQGVYGWLNA-DN   193
SA          PWNYPLLQASWKIAPALATGCSLVMKPSEITPLTTIRVFELMEEVGFPKGTINLILGAGS   214
TT          PWNAPLMLSTWRIAPALAFGNTVVLKPAEWSPFTATKLAEILKEADLPPGVFNLVQGFGE   230
ST          PFNFPINSFAHKVAPAIAVGNSVVVKPSISTPLSAIEMKKILVEAGLPDSAVRIVTGYSN   199
            *:* *:        .* :  *   ::* :        :  : : : ... * .     :   .

S2-ALDH     KRSTALVEDVRIKGISLTGSEGAGASMAEAAGKNLKRSVLELGGSDAFIILADADIDKTV   247
FF          KLVEQVIEDKNIVAITLTGSDPAGRSVASIAGKNLKKTVMELGGSDAYVVLDDVDLEKAT   248
EC          DGVSQMIKDSRIAAVTVTGSVRAGAAIGAQAGAALKKCVLELGGSDPFIVLNDADLELAV   253
SA          EVGDVMSGHKEVDLVSFTGGIETGKHIMKNAANNVTNIALELGGKNPNIIFDDADFELAV   274
TT          EAGAALVAHPLVPLLTLTGETETGKIVMRNAADHLKRLSPELGGKSPALVFADADLERAL   290
ST          EIGDELITHPLVGLITLTGSTQTGLAIASKAVSLGKRIIMELGGSDPIIVLEDANIDRAS   259
            .    :  .  :  ::.**    :*  :    *     ..    ****..  ::: *.::: :

S2-ALDH     DLAIFGRMNNNGQSCVASKRFIAVEAIADEFLEKFTAKVAALKVGDPMDPSTDVGPLSSE   307
FF          DLATLGRLQNNGQTCIAAKRFVVLEAIYDDFLALFTKKMKAAKMGEPTNEDTYYGPMARK   308
EC          KAAVAGRYQNTGQVCAAAKRFIIEEGIASAFTERFVAAAAALKMGDPRDEENTLGPMARF   313
SA          DQALNGGYFHAGQVCSAGSRILVQNSIKDKFEQALIDRVKKIKLGNGFDADTEMGPVIST   334
TT          DAVVFQIFSFNGERCTASSRLLVEEKIFEDFVGKVVERARAIRVGHPLDPETEVGPLIHP   350
ST          SIAVRARYEYAGQNCNAGKRIIVREEIYDKFVKAFKEKVKALKVGDPLDESTDIGPVINQ   319
            . .         *: * *..*::  : * . *     .        ::*.  : ..  **:

S2-ALDH     EALQGLLEQVKKFEAAGATVVVGGKRASK--------EGAYMQPTLLTNLKRGEPTFYEE   359
FF          DLRDELHEQVLKTVGQGGKLVLGGEIPNQ--------KGAYYPATILADLKPGMEGFDNE   360
EC          DLRDELHHQVEKTLAQGARLLLGGEKMAG--------AGNYYPPTVLANVTPEMTAFREE   365
SA          EHRNKIESYMDVAKAEGATIAVGGKRPDR----DDLKDGLFFEPTVITNCDTSMRIVQEE   390
TT          EHLQRVLGYVEAGKREGARLLVGGERAKTSFRGEDLSRGNYLLPTVFV-GENHMKIAQEE   409
ST          ESVEKLNKALEDAQSKGGNVEVLNK-GPE--------TGYFFPLSLVTNPSLDMLVLKTE   370
            :  : :    :       *. : : .:              * :    ::..             *

S2-ALDH     LFGPVASFYKVKDEQEAIDLANDSPFGLGGSIYTKDIKNAIKIADQIDSGMVFINSPTW-   418
FF          LFGPVASVIRAKDDDDAIALANNSQYGLGSGVLTSNNERGEKIALQLEAGSSFVNKLVV-   419
EC          MFGPVAAITIAKDAEHALELANNSEFGLSATIFTTDETRARQMAARLECGGVFINGYCA-   424
SA          VFGPVVTVEGFETEQEAIQLANDSIYGLAGAVFSKDIGKAQRVANKLKLGTVWINDFHP-   449
TT          IFGPVLVAIPFKDEEEALRKANDTKYGLAAYVFTRDLERAHRLALELEAGMVYLNSHNV-   468
ST          IFGPIAPIVSVKSDEEAINIANSTEYGLQSAIFSNDVNRALKIAKELKFGAIIINDSTRL   430
            :***:       :  :.*:  **.: :** . : : :  .. ::* .:. *    :*

S2-ALDH     TQADLPFGGTKRSGYGRELSEQGIQEFVNKKLIRVS-----------        454
FF          SDPRLPFGGVKKSGYGRELAAYGIREFVNTKTIWID-----------        455
EC          SDARVAFGGVKKSGFGRELSHFGLREFCNIQTVWKDRI---------        462
SA          YFAQAPWGGYKQSGIGRELGKEGLEEYIVSKHILTNTNPQLVNWFSK        496
TT          RHLPTPFGGVKG-----------------------------------        480
ST          RWDSLPFGGFKKTGIGREGVRDTMLEMTENKLIAITLL---------        468
                   :** *
```

Figure 3S. Multiple alignment of S2-ALDH primary structure with the homologous enzymes from the psychrophilic *Flavobacterium frigidarium* [WP_026709111], mesophilic *Escherichia coli* [WP_089574674] and *Staphylococcus aureus* [WP000421701], thermophilic *Thermus thermophilus* [WP_011173038] and hyperthermophilic *Sulfurisphaera tokadaii str. 7* [BAB65021]; Identical (stars) and conserved (dots) residues from all enzymes; active site residues (blue); NAD^+ cofactor binding site residues (grey shade); oligomerization domain (box).

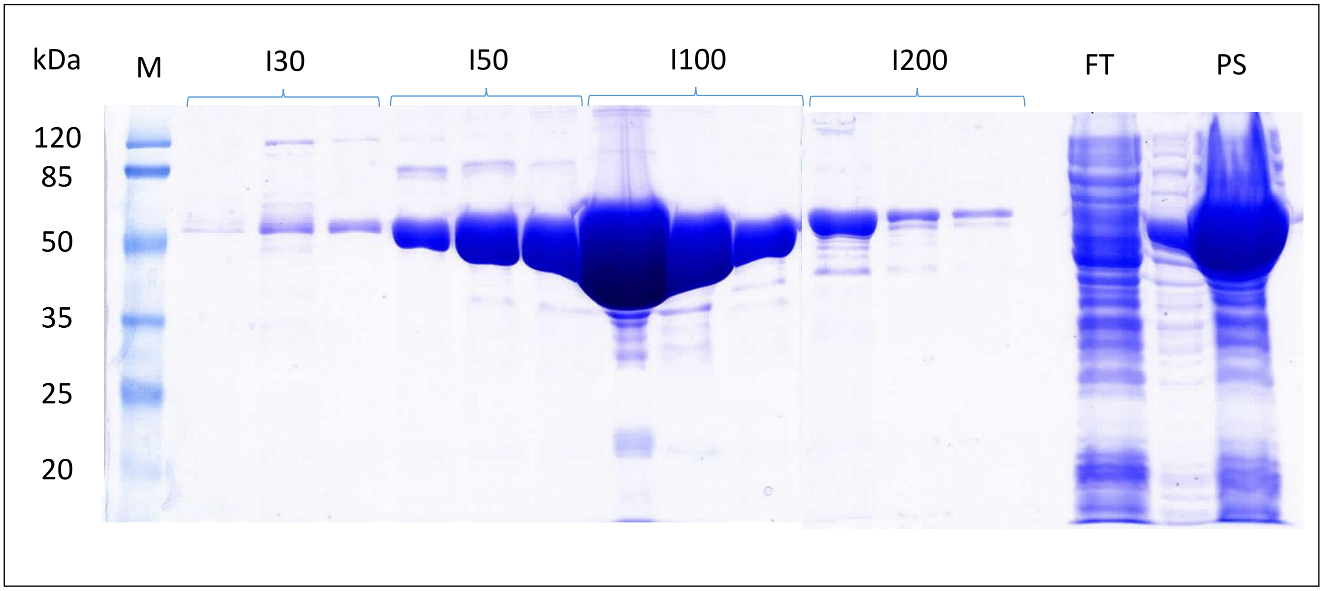

Figure 4S. Purification of recombinant ALDH-S2 by Ni2+-NTA affinity chromatography. Elution fractions analyzed by SDS-PAGE (M): standard molecular mass proteins (Thermo Scientific); (I30 mM – I200 mM): imidazole concentration in TN buffer (100 mM TrisHCl, pH 8, 200 mM NaCl)

Characterization of CNT electrodes

CNT electrodes are carbon electrodes functionalized with multi wall carbon nanotubes with carboxylic end groups (the level of -COOH functionalization is around 5%) with a characteristic Raman spectrum (Panel A). The G band of the Raman spectra is shown at around 1580cm^{-1} providing information about the ratio of sp2 bonds. Additionally D band shown at around 1300cm^{-1} is providing information about the quantity of sp3 bonds, related to the defects in the carbon nanotubes structure. Due to the intensity of both bands we can ensure from a qualitative analysis that the nanotubes used in this work are highly functionalized.

The CNTs have an average diameter of 10 nm and an average length of 1.5 µm. The average thickness of the CNT layer is 18 µm and the roughness Ra is 1µm as revealed by Scanning Electron Microscopy imaging (Panel B). The high surface to volume ratio and the morphology of the CNT layer favor the sensitive electrochemical detection of NADH. Moreover, the CNTs have an electrocatalytic effect for the direct oxidation of NADH as the anodic peak potential in cyclic voltammetry is decreased by 36 mV compared to bare carbon electrodes, to 0.518V (Panel C).

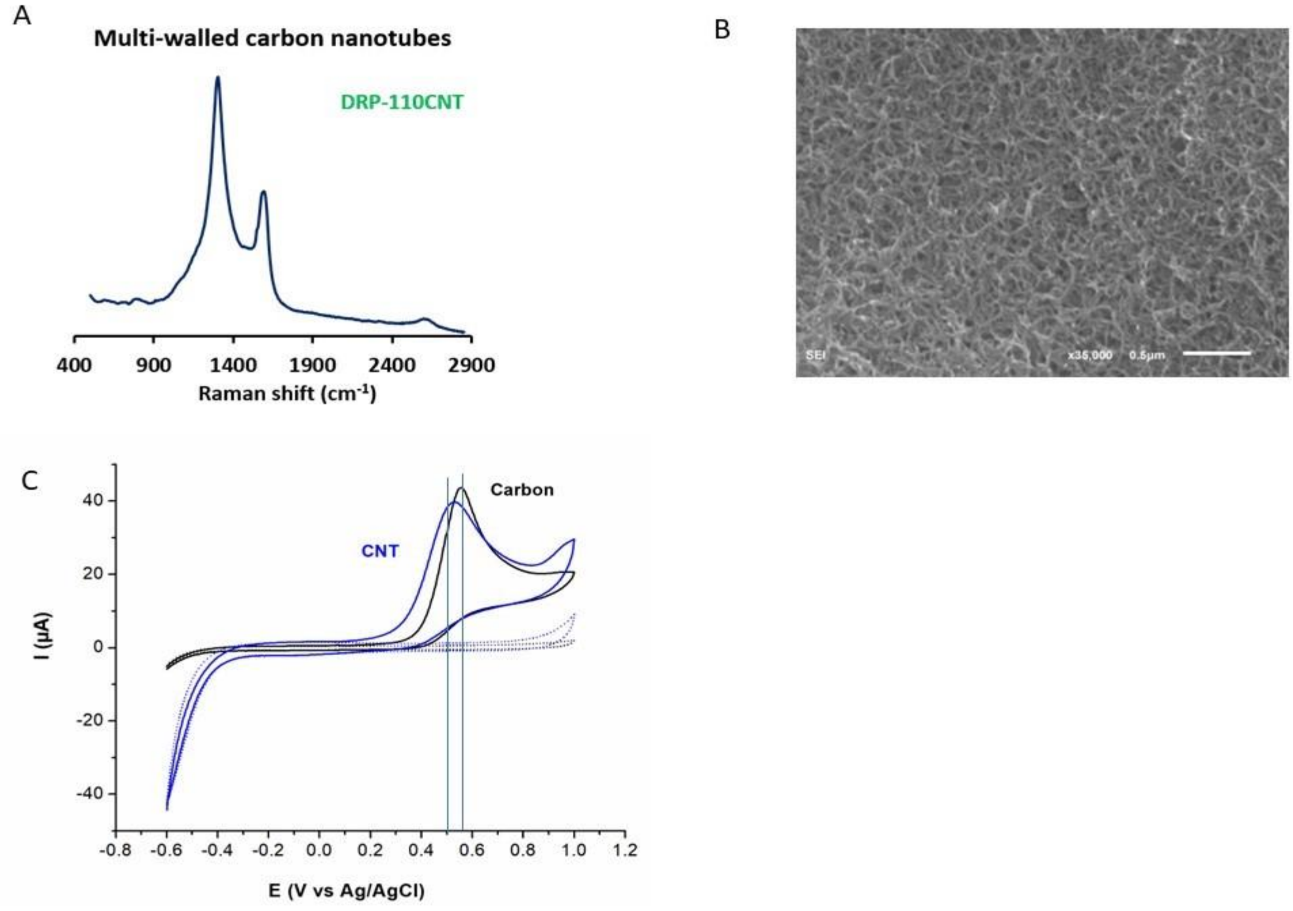

Figure 5S. Characterization of CNT electrodes used as NADH detectors in the electrochemical assays based on S2-ALDH. **(A)** Raman spectrum. **(B)** Scanning electron microscopy (SEM) image. **(C)** Cyclic voltammograms recorded at a scan speed of 100 mV/s in the range from -0.6 to 1.0 V, in PBS buffer pH 7.4 in the absence (dashed lines) and the presence of 2 mM NADH, respectively (solid lines) with CNT electrodes (blue), and with bare carbon electrodes from the same manufacturer (black).

Table 2S. Comparison of the performances of the S2-ALDH-based amperometric assay using screen-printed CNT electrodes and other electrochemical sensors for NADH

Description	Applied potential (V)	Detection limit (μM)	Linear range (μM)	Sensitivity	Reference
Meldola Blue-electrochemically reduced graphene oxide/carbon nanofiber (screen-printed)	+ 0.05	0.3	1–300<br>300-700	80.0±2.5 μA cm-2 mmol-1 L<br><br>65.1 ± 12.0 μA cm-2 $mmol^{-1}$ L	Titoiu et al., 2018
MWCNT (microwave oxidized) deposited on screen printed electrode	0.27<br>(vs Ag/AgCl)	1	4-35	Not mentioned	Blandón-Naranjo, et al., 2018
Aluminium hydroxide/iron hydroxide/MWCNTs nanocomposite film-modified electrode	0.15<br>(vs Ag/AgCl, PBS)	0.3	0.5–220	0.02 μA/μM	Wang et al., 2020
Glassy carbon/CNT-Chitosan	0.4<br>(vs Ag/AgCl)	3	5-300	130 ± 6 mA M^{-1} cm^{-2}	Zhang et al., 2004
Glassy carbon/MWCNTs, acid microwaved or water-boiled	-0.05<br>(vs Ag/AgCl)	2 (acid microwaved CNT)<br><br>4 (water boiled CNT)	10-100	12 mA M^{-1} cm^{-2}<br><br>3.2 mA M^{-1} cm^{-2} (water boiled CNT)	Wooten et al., 2010
Screen printed carbon electrode/reduced graphene oxide	0.53<br>(vs Ag/AgCl pseudo reference electrode)	5	Not mentioned	88.5 mA M^{-1} cm^{-2}	Cinti et al., 2015
Glassy carbon/<br>Carbon nanocages	0.2<br>(vs Ag/AgCl)	0.34	0.34-450	15.78 μA μM^{-1} cm^{-2}	Mao et al., 2021
Glassy carbon /MWCNT/ poly(caffeic acid)	0.1<br>(vs Ag/AgCl)	0.12	0.5-1270	85.7 μA mM^{-1} cm^{-2}	Rebis et al., 2021
CNT SPE	0.5<br>(vs Ag pseudo reference electrode)	10	12.5-250	1.15 ± 0.16<br>μA L/ $mmol^{-1}$	This work

Optimization of wine pre-treatment for the electrochemical assays based on S2-ALDH (details)

The efficiency of the treatment to remove the phenolic compounds was determined using the electrochemical signal of a red wine filtered through various cartridges. For that, 25 µL of pre-treated red wine sample were added in the cell containing 75 µL 0.2 M phosphate buffer pH 7.5. The current intensity was recorded at 100 s, 400 s and 600 s. A model wine solution, filtered in the same way, was also analyzed. The investigated samples were: unfiltered wine (i), wine filtered through a cartridge (1 mL plastic syringe) filled with 0.15 g PVPP (ii), wine filtered through a cartridge filled with 50 mg activated charcoal (iii) and wine filtered through a cartridge filled with 0.15 g PVPP and 25 mg activated charcoal (iv). All filtration cartridges were preconditioned before use by passing 1 ml of 0.2 M phosphate buffer pH 7.5, followed by drying at room temperature. An additional test was made with wine and model wine solution filtered through the cartridge containing both PVPP and activated charcoal. These were tested with both bare and "blocked" carbon nanotube electrodes. The electrodes were "blocked" by a 30 minutes incubation with a 10 mg/ml solution of bovine serum albumin in PBS buffer pH 7.4, to prevent the non-specific adsorption of wine phenols.

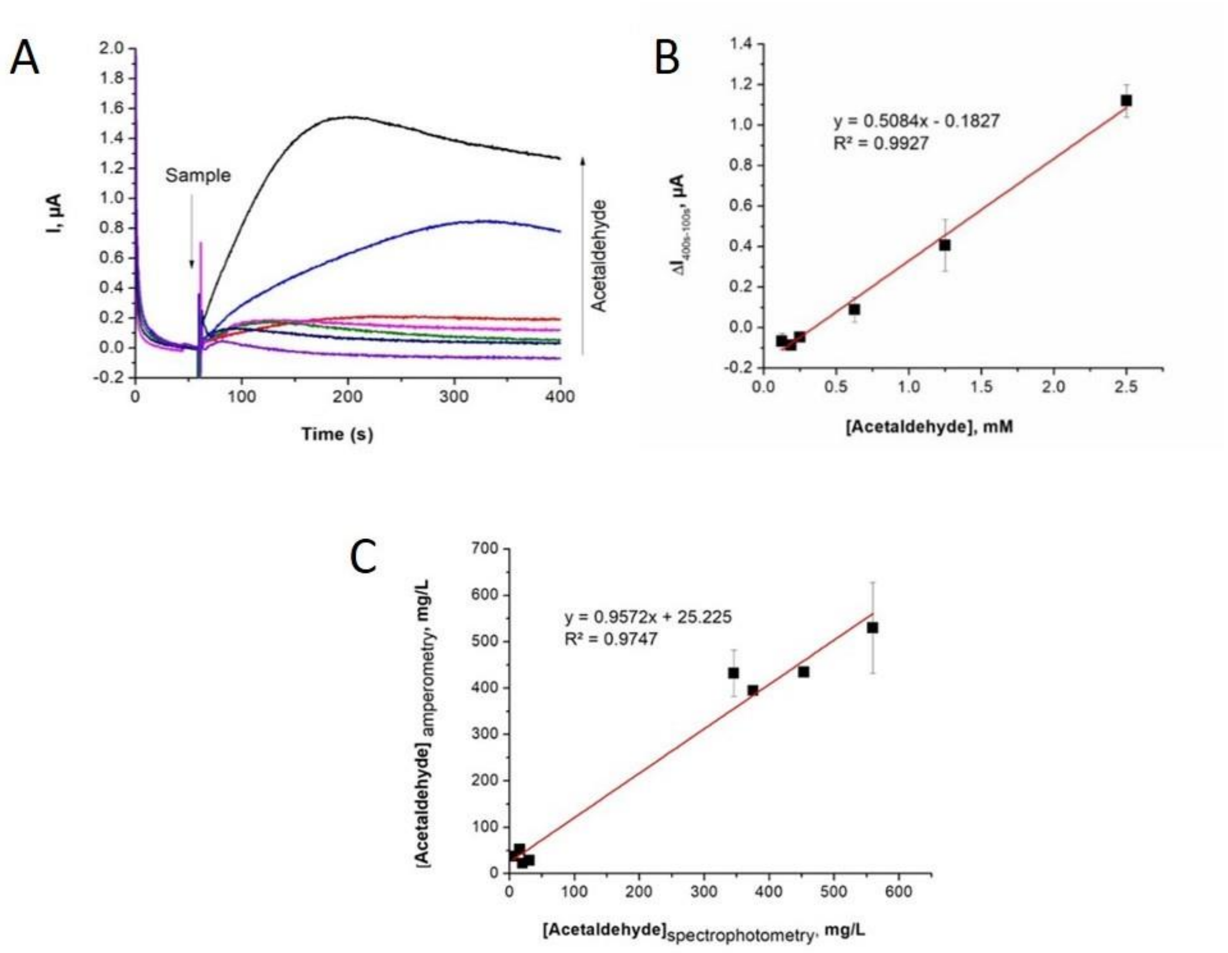

Figure 6S. Calibration for acetaldehyde detection in model wine solution at 17°C**. (A)** Plots of current intensity as a function of time for the determination of acetaldehyde in wine at 0.5 V and 17° C for solutions of acetaldehyde in model wine solution of different concentrations (0; 0.5; 0.75, 1, 2.5, 5 and 10 mM); **(B)** Corresponding calibration curve. **(C)** Correlation between the electrochemical assay based on ALDS2 and the reference spectrophotometric method for acetaldehyde for a set of 8 wines.
